# Are innovation and new technologies in precision medicine paving a new era in patients centric care?

**DOI:** 10.1186/s12967-019-1864-9

**Published:** 2019-04-05

**Authors:** Attila A. Seyhan, Claudio Carini

**Affiliations:** 1 Department of Pathology and Laboratory Medicine, Division of Biology and Medicine, Brown University, Providence, RI 02903 USA; 2 Fox Chase Cancer Center, Temple University Temple Health, Philadelphia, PA 19111 USA; 3 School of Cancer and Pharmaceutical Sciences, Faculty of Life Sciences & Medicine, King’s College London, London, SE1 8WA UK

**Keywords:** Precision medicine, Personalized medicine, Biomarkers, Modeling and simulation, Digital biomarkers, Artificial intelligence, Machine learning, Deep phenotyping, Cancer, Immuno-oncology, Autoimmune and inflammatory diseases, Diabetes, Genetics, Epigenetics, Genomics, Transcriptomics, microRNAs, miRNAs, Proteomics

## Abstract

Healthcare is undergoing a transformation, and it is imperative to leverage new technologies to generate new data and support the advent of precision medicine (PM). Recent scientific breakthroughs and technological advancements have improved our understanding of disease pathogenesis and changed the way we diagnose and treat disease leading to more precise, predictable and powerful health care that is customized for the individual patient. Genetic, genomics, and epigenetic alterations appear to be contributing to different diseases. Deep clinical phenotyping, combined with advanced molecular phenotypic profiling, enables the construction of causal network models in which a genomic region is proposed to influence the levels of transcripts, proteins, and metabolites. Phenotypic analysis bears great importance to elucidat the pathophysiology of networks at the molecular and cellular level. Digital biomarkers (BMs) can have several applications beyond clinical trials in diagnostics—to identify patients affected by a disease or to guide treatment. Digital BMs present a big opportunity to measure clinical endpoints in a remote, objective and unbiased manner. However, the use of “omics” technologies and large sample sizes have generated massive amounts of data sets, and their analyses have become a major bottleneck requiring sophisticated computational and statistical methods. With the wealth of information for different diseases and its link to intrinsic biology, the challenge is now to turn the multi-parametric taxonomic classification of a disease into better clinical decision-making by more precisely defining a disease. As a result, the big data revolution has provided an opportunity to apply artificial intelligence (AI) and machine learning algorithms to this vast data set. The advancements in digital health opportunities have also arisen numerous questions and concerns on the future of healthcare practices in particular with what regards the reliability of AI diagnostic tools, the impact on clinical practice and vulnerability of algorithms. AI, machine learning algorithms, computational biology, and digital BMs will offer an opportunity to translate new data into actionable information thus, allowing earlier diagnosis and precise treatment options. A better understanding and cohesiveness of the different components of the knowledge network is a must to fully exploit the potential of it.

## Introduction

Today, the practice of medicine remains largely empirical; physicians generally rely on patterns matching to establish a diagnosis based on a combination of the patients’ medical history, physical examination, and laboratory data. Thus, a given treatment is often based on physicians past experience with similar patients. One consequence of this is that a blockbuster gets prescribed for a “typical patient” with a specific disease. According to this paradigm, treatment decision is driven by trial and error and the patient occasionally becomes the victim of unpredictable side effects, or poor or no efficacy for a drug that theoretically works in some people affected by that specific disease.

Greater use of BMs [1, 2] and companion diagnostics (CDX) can now enable a shift from empirical medicine to precision medicine (PM) (the right medicine, for the right patient, at the right dose, at the right time). It is conceivable that, in the immediate future, physicians will be moving away from the concept of “one size fits all” and shift instead to PM.

It is generally known that the response of a specific treatment varies across the heterogeneity of a population with good and poor responders. Patients and treatment response differ because of variables like genetic predisposition, heterogeneity of the cohorts, ethnicity, slow vs. fast metabolizers, epigenetic factors, early vs. late stage of the disease. These parameters have an effect on whether a given individual will be a good or poor responder to a specific treatment.

The goal of PM is to enable clinicians to quickly, efficiently and accurately predict the most appropriate course of action for a patient. To achieve this, clinicians are in need of tools that are both compat-ible with their clinical workflow and economically feasible. Those tools can simplify the process of managing the biological complexity that underlies human diseases. To support the creation and refinement of those tools, a PM ecosystem is in continuous development and is the solution to the problem. The PM ecosystem is beginning to link and share information among clinicians, laboratories, research enterprises, and clinical-information-system developers. It is expected that these efforts will create the foundation of a continuously evolving health-care system that is capable of significantly accelerating the advancement of PM technologies.

Precision medicine highlights the importance of coupling established clinical indexes with molecular profiling in order to craft diagnostic, prognostic and therapeutic strategies specific for the needs of each group of patients. A correct interpretation of the data is a must for the best use of the PM ecosystem. The PM ecosystem combines omics and clinical data to determine the best course of action to be taken for each specific patient group.

Currently, a drug gets approved after a lengthy regulatory process. One way to address this problem, is to focus on selected group of patients thus, Phase III clinical studies can be conducted with a small group of patients rather than thousands and thousands of patients typically needed for the Phase III studies. This approach should potentially guarantee a more rapid and expeditious way to perform drug development of next-generation pharmacotherapy. A narrower focus on a specific patient’s group at the stage of the regulatory approval process should facilitate streamlining the regulatory approval resulting in a greater clinical and economic success.

The shift towards a deeper understanding of disease based on molecular biology will also inevitably lead to a new, more precise disease’s classification, incorporating new molecular knowledge to generate a new taxonomy. This change will result in a revised classification of intrinsic biology, leading to revisions of diseases signs and symptoms. For this change to occur, however, larger data bases, accessible to all, will be needed that dynamically incorporate new information.

The emerging use of personalized laboratory medicine makes use of a multitude of testing options that can more precisely pinpoint management needs of individual groups of patients. PM seeks to dichotomize patient populations in those who might benefit from a specific treatment (responders) and those for whom a benefit is improbable (non-responders). Defining cut-off points and criteria for such a dichotomy is difficult. Treatment recommendations are often generated using algorithms based on individual somatic genotype alterations. However, tumors often harbor multiple drivers’ mutations (owing to intra- and inter-tumoral heterogeneity). Physicians, therefore, need to combine different streams of evidence to prioritize their choice of treatment. The implementation of PM often relies on a fragmented landscape of evidences making hard for physicians to select among different diagnostic tools and treatment options.

**Fig. 1:**
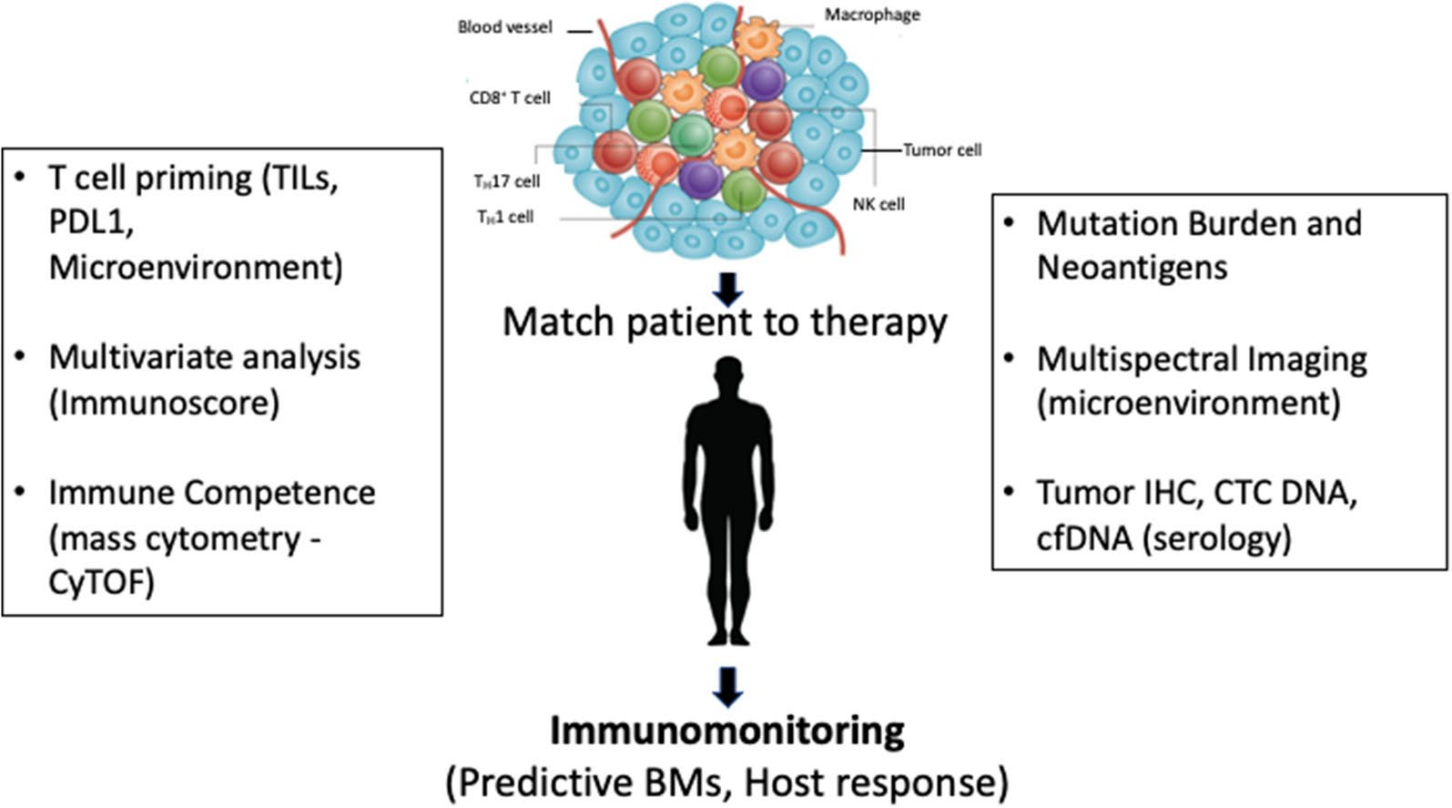
Critical checkpoints for host and tumor profiling. A multiplexed biomarker approach is highly integrative and includes both tumor- and immune-related parameters assessed with both molecular and image-based methods for individualized prediction of immunotherapy response. By assessing patient samples continuously one can collect a dynamic data on tissue-based parameters, such as immune cell infiltration and expression of immune checkpoints, and pathology methods. These parameters are equally suited for data integration with molecular parameters. TILs: tumor-infiltrating lymphocytes. PD-L1: programmed cell death-ligand 1. Immunoscore: a prognostic tool for quantification of in situ immune cell infiltrates. Immunocompetence: body’s ability to produce a normal immune response following exposure to an antigen (tumor drawing has been adapted from [42])

**Fig. 2:**
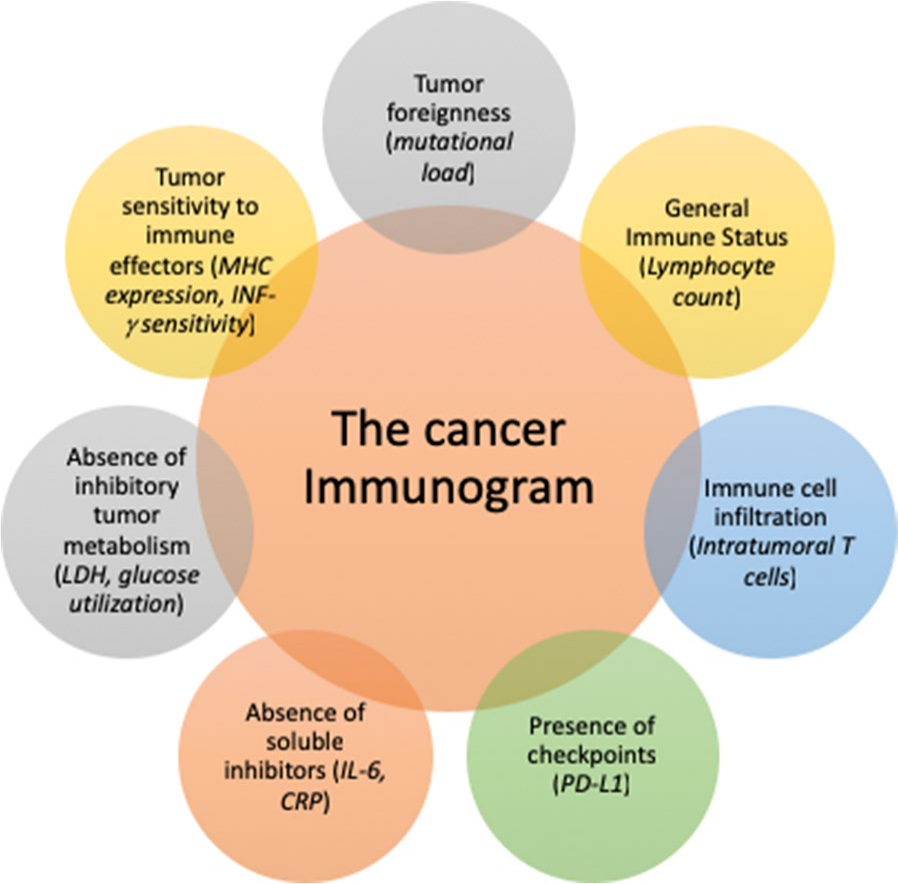
The cancer immunogram. The schema depicts the seven parameters that characterize aspects of cancer-immune interactions for which biomarkers have been identified or are plausible. Italics represent those potential biomarkers for the different parameters (adapted from [4])

In the case of cancer immunotherapy, predictive biomarkers (BM) for immunotherapy differ from the traditional BM used for targeted therapies. The complexity of the tumor microenvironment (TME), the immune response and molecular profiling requires a more holistic approach than the use of a single analyte BM [3]. To cope with this challenge, researchers have adopted multiplexing approach, where multiple BMs are used to empower more accurate patient stratification [4]. To select specific patient’s groups for immunotherapy, histological analysis now include concomitant analysis of immuno-oncology BMs, such as PD-L1 and immune cell infiltrates (Fig. 1) as well as more comprehensive immune and tumor-related pathways (the “Cancer Immunogram”) (Fig. 2) [4, 5]. In the case of cancer immunotherapy, multiplexed immunoprofiling generating a comprehensive biomarker dataset that can correlated with clinical parameters is key for the success of PM.

### Patient stratification for precision medicine

In traditional drug development, patients with a disease are enrolled randomly to avoid bias, using an “all comers” approach with the assumption that the enrolled patients are virtually homogeneous. The reason for random enrollment is to ensure a wide representation of the general population. In reality, we never perform clinical trials for randomly selected patients, but rather we apply various types of enrichments to patients’ enrolment by applying specific inclusion and exclusion criteria. Despite all of those efforts to increase the enrichment, the population that ultimately gets selected for the study can be rather heterogeneous with respect to drug-metabolizing capabilities, environmental conditions (e.g. diet, smoking habit, lifestyle etc.), or previous exposure to medication(s) as well as individuals genetic and epigenetic make-up. By using BMs to better characterize molecular, genetic, and epigenetic makeup of patients, drug developers have been trying to establish a more objective approach.

The use of patient stratification is to separate probable responders from non-responders. A prospective stratification can result in a smaller and shorter clinical study compared to those needed for randomly selected patients.

Minimally, stratification can speed up approval for drug candidates intended for a subset of patients, while leaving the door open for further tests and market expansion in the more heterogeneous population of patients. Maximally, it can unmask a useful therapeutic agent that otherwise would be lost in the noise generated by the non-responders, as was the case for instance of trastuzumab and gefitinib [6].

Thus, clinical trials could be shorter, given a quicker determination on the efficacy of the new molecular entity. Today, the major focus of research is to identify the molecular causes of differential therapeutic responses across patient populations. It is now clear that patients affected by a disease show significant response heterogeneity to a given treatment. Advances in understanding the mechanisms underlying diseases and drug response are increasingly creating opportunities to match patients with therapies that are more likely to be efficacious and safer.

Furthermore, patient stratification has a considerable economic impact on the model of the pharmaceutical industry. By identifying the populations likely to benefit from a new therapy, drug development costs will be reduced and the risk of treating non-responders will be minimized. Advances in “omics” technologies (e.g. epigenomics, genomics, transcriptomics, proteomics, metabolomics, etc.), also called, systems-based approach [7], are now utilized to identify molecular targets including BMs [1, 2] that can reveal the disease state or the ability to respond to a specific treatment, thus providing scientists and clinicians to generate a learning dataset consisting of molecular insights of the disease pathogenesis.

A search of the relevant literature will reveal an abundance of publications related to BMs [8]. However, as previously reported by Poste in 2011 [9] more than 150,000 articles have described thousands of BMs however, only approximately 100 BMs are routinely used in the clinical practice. As to date, over 355 new non-traditional BMs (i.e. pharmacogenomic BM-drug pairs) have been described in drug labels (www.fda.gov/drugs/scienceresearch/ucm572698.htm). Table 1 lists 355 pharmacogenomic BMs as of Dec. 2018, linked to drugs with pharmacogenomic information found in the drug labeling (Drugs@FDA; https://www.fda.gov/drugs/scienceresearch/ucm572698.htm). Those BMs include germline or somatic gene variants (i.e. polymorphisms, mutations), functional deficiencies with a genetic etiology, altered gene expression signatures, and chromosomal abnormalities. The list also includes selected protein BMs that are used to select treatments for specific patient’s groups.

**Table 1:** Pharmacogenomic BMs in drug labeling

Drug	Therapeutic area	Biomarker	Labeling sections
Abacavir	Infectious Diseases	HLA-B	Boxed warning, dosage and administration, contraindications, warnings and precautions
Abemaciclib (1)	Oncology	ESR (Hormone receptor)	Indications and usage, adverse reactions, clinical studies
Abemaciclib (2)	Oncology	ERBB2 (HER2)	Indications and usage, adverse reactions, clinical studies
Ado-Trastuzumab Emtansine	Oncology	ERBB2 (HER2)	Indications and usage, warnings and precautions, adverse reactions, clinical pharmacology, clinical studies
Afatinib	Oncology	EGFR	Indications and usage, dosage and administration, adverse reactions, clinical studies
Alectinib	Oncology	ALK	Indications and usage, dosage and administration, adverse reactions, clinical pharmacology, clinical studies
Amitriptyline	Psychiatry	CYP2D6	Precautions
Anastrozole	Oncology	ESR, PGR (Hormone receptor)	Indications and usage, adverse reactions, drug interactions, clinical studies
Arformoterol (1)	Pulmonary	UGT1A1	Clinical pharmacology
Arformoterol (2)	Pulmonary	CYP2D6	Clinical pharmacology
Aripiprazole	Psychiatry	CYP2D6	Dosage and administration, use in specific populations, clinical pharmacology
Aripiprazole lauroxil	Psychiatry	CYP2D6	Dosage and administration, use in specific populations, clinical pharmacology
Arsenic trioxide	Oncology	PML-RARA	Indications and usage
Atezolizumab	Oncology	CD274 (PD-L1)	Indications and usage, adverse reactions, clinical pharmacology, clinical studies
Atomoxetine	Psychiatry	CYP2D6	Dosage and administration, warnings and precautions, adverse reactions, drug interactions, clinical pharmacology
Ascorbic acid, PEG-3350, potassium chloride, sodium ascorbate, sodium chloride, and sodium sulfate	Gastroenterology	G6PD	Warnings and precautions
Avatrombopag (1)	Hematology	F2 (Prothrombin)	Warnings and precautions
Avatrombopag (2)	Hematology	F5 (Factor V Leiden)	Warnings and precautions
Avatrombopag (3)	Hematology	PROC	Warnings and precautions
Avatrombopag (4)	Hematology	PROS1	Warnings and precautions
Avatrombopag (5)	Hematology	SERPINC1 (Antithrombin III)	Warnings and precautions
Avelumab	Oncology	CD274 (PD-L1)	Clinical studies
Azathioprine	Rheumatology	TPMT	Dosage and administration, warnings, precautions, drug interactions, adverse reactions, clinical pharmacology
Belinostat	Oncology	UGT1A1	Dosage and administration, clinical pharmacology
Binimetinib (1)	Oncology	BRAF	Indications and usage, dosage and administration, warnings and precautions, adverse reactions, use in specific populations, clinical studies
Binimetinib (2)	Oncology	UGT1A1	Clinical pharmacology
Blinatumomab	Oncology	BCR-ABL1 (Philadelphia chromosome)	Indications and usage, clinical studies
Boceprevir	Infectious Diseases	IFNL3 (IL28B)	Clinical pharmacology
Bosutinib	Oncology	BCR-ABL1 (Philadelphia chromosome)	Indications and usage, adverse reactions, use in specific populations, clinical studies
Brentuximab vedotin	Oncology	ALK	Clinical studies
Brexpiprazole	Psychiatry	CYP2D6	Dosage and administration, use in specific populations, clinical pharmacology
Brigatinib	Oncology	ALK	Indications and usage, adverse reactions, clinical studies
Brivaracetam	Neurology	CYP2C19	Clinical pharmacology
Busulfan	Oncology	BCR-ABL1 (Philadelphia chromosome)	Clinical studies
Cabozantinib	Oncology	RET	Clinical studies
Capecitabine	Oncology	DPYD	Warnings and precautions, patient counseling information
Carbamazepine (1)	Neurology	HLA-B	Boxed warning, warnings, precautions
Carbamazepine (2)	Neurology	HLA-A	Warnings
Carglumic Acid	Inborn errors of metabolism	NAGS	Indications and usage, warnings and precautions, use in specific populations, clinical pharmacology, clinical studies
Cariprazine	Psychiatry	CYP2D6	Clinical pharmacology
Carisoprodol	Rheumatology	CYP2C19	Use in specific populations, clinical pharmacology
Carvedilol	Cardiology	CYP2D6	Drug interactions, clinical pharmacology
Celecoxib	Rheumatology	CYP2C9	Dosage and administration, use in specific populations, clinical pharmacology
Ceritinib	Oncology	ALK	Indications and usage, dosage and administration, adverse reactions, clinical studies
Cerliponase alfa	Inborn errors of metabolism	TPP1	Indications and usage, use in specific populations, clinical studies
Cetuximab (1)	Oncology	EGFR	Indications and usage, dosage and administration, warnings and precautions, adverse reactions, clinical studies
Cetuximab (2)	Oncology	RAS	Indications and usage, dosage and administration, warnings and precautions, adverse reactions, clinical studies
Cevimeline	Dental	CYP2D6	Precautions
Chloroquine	Infectious diseases	G6PD	Precautions
Chlorpropamide	Endocrinology	G6PD	Precautions
Cisplatin	Oncology	TPMT	Adverse reactions
Citalopram (1)	Psychiatry	CYP2C19	Dosage and administration, warnings, clinical pharmacology
Citalopram (2)	Psychiatry	CYP2D6	Clinical pharmacology
Clobazam	Neurology	CYP2C19	Dosage and administration, use in specific populations, clinical pharmacology
Clomipramine	Psychiatry	CYP2D6	Precautions
Clopidogrel	Cardiology	CYP2C19	Boxed warning, warnings and precautions, clinical pharmacology
Clozapine	Psychiatry	CYP2D6	Dosage and administration, use in specific populations, clinical pharmacology
Cobimetinib	Oncology	BRAF	Indications and usage, dosage and administration, adverse reactions, clinical studies
Codeine	Anesthesiology	CYP2D6	Boxed warning, warnings and precautions, use in specific populations, patient counseling information
Crizotinib (1)	Oncology	ALK	Indications and usage, dosage and administration, adverse reactions, use in specific populations, clinical pharmacology, clinical studies
Crizotinib (2)	Oncology	ROS1	Indications and usage, dosage and administration, adverse reactions, use in specific populations, clinical studies
Dabrafenib (1)	Oncology	BRAF	Indications and usage, dosage and administration, warnings and precautions, adverse reactions, clinical pharmacology, clinical studies, patient counseling information
Dabrafenib (2)	Oncology	G6PD	Warnings and precautions, adverse reactions, patient counseling information
Dabrafenib (3)	Oncology	RAS	Dosage and administration, warnings and precautions
Daclatasvir	Infectious diseases	IFNL3 (IL28B)	Clinical studies
Dapsone (1)	Dermatology	G6PD	Warnings and precautions, use in specific populations
Dapsone (2)	Dermatology	Nonspecific (Congenital Methemoglobinemia)	Warnings and precautions
Dapsone (3)	Infectious Diseases	G6PD	Precautions, adverse reactions, overdosage
Darifenacin	Urology	CYP2D6	Clinical pharmacology
Dasabuvir, ombitasvir, paritaprevir, and ritonavir	Infectious Diseases	IFNL3 (IL28B)	Clinical studies
Dasatinib	Oncology	BCR-ABL1 (Philadelphia chromosome)	Indications and usage, dosage and administration, warnings and precautions, adverse reactions, clinical studies
Denileukin diftitox	Oncology	IL2RA (CD25 antigen)	Indications and usage, warnings and precautions, clinical studies
Desipramine	Psychiatry	CYP2D6	Precautions
Desflurane	Anesthesiology	Nonspecific (Genetic Susceptibility to Malignant Hyperthermia)	Contraindications
Desvenlafaxine	Psychiatry	CYP2D6	Clinical pharmacology
Deutetrabenazine	Neurology	CYP2D6	Dosage and administration, warnings and precautions, use in specific populations, clinical pharmacology
Dexlansoprazole	Gastroenterology	CYP2C19	Drug interactions, clinical pharmacology
Dextromethorphan and quinidine	Neurology	CYP2D6	Warnings and precautions, clinical pharmacology
Diazepam	Neurology	CYP2C19	Clinical pharmacology
Dinutuximab	Oncology	MYCN	Clinical studies
Dolutegravir	Infectious Diseases	UGT1A1	Clinical pharmacology
Doxepin (1)	Psychiatry	CYP2D6	Clinical pharmacology
Doxepin (2)	Psychiatry	CYP2C19	Clinical pharmacology
Dronabinol	Gastroenterology	CYP2C9	Use in specific populations, clinical pharmacology
Drospirenone and ethinyl estradiol	Gynecology	CYP2C19	Clinical pharmacology
Duloxetine	Psychiatry	CYP2D6	Drug interactions
Durvalumab	Oncology	CD274 (PD-L1)	Clinical pharmacology, clinical studies
Efavirenz	Infectious diseases	CYP2B6	Clinical pharmacology
Elbasvir and grazoprevir	Infectious diseases	IFNL3 (IL28B)	Clinical studies
Eliglustat	Inborn errors of metabolism	CYP2D6	Indications and usage, dosage and administration, contraindications, warnings and precautions, drug interactions, use in specific populations, clinical pharmacology, clinical studies
Elosulfase	Inborn errors of metabolism	GALNS	Indications and usage, warnings and precautions, use in specific populations, clinical pharmacology, clinical studies
Eltrombopag (1)	Hematology	F5 (Factor V Leiden)	Warnings and precautions
Eltrombopag (2)	Hematology	SERPINC1 (Antithrombin III)	Warnings and precautions
Enasidenib	Oncology	IDH2	Indications and usage, dosage and administration, clinical pharmacology, clinical studies
Encorafenib	Oncology	BRAF	Indications and usage, dosage and administration, warnings and precautions, adverse reactions, use in specific populations, clinical pharmacology, clinical studies
Enflurane	Anesthesiology	Nonspecific (genetic susceptibility to malignant hyperthermia)	Contraindications
Erlotinib	Oncology	EGFR	Indications and usage, dosage and administration, adverse reactions, clinical studies
Erythromycin and sulfisoxazole	Infectious Diseases	G6PD	Precautions
Escitalopram (1)	Psychiatry	CYP2D6	Drug interactions
Escitalopram (2)	Psychiatry	CYP2C19	Adverse reactions
Esomeprazole	Gastroenterology	CYP2C19	Drug interactions, clinical pharmacology
Eteplirsen	Neurology	DMD	Indications and usage, adverse reactions, use in specific populations, clinical studies
Everolimus (1)	Oncology	ERBB2 (HER2)	Indications and usage, dosage and administration, warnings and precautions, adverse reactions, drug interactions, use in specific populations, clinical studies
Everolimus (2)	Oncology	ESR (hormone receptor)	Indications and usage, dosage and administration, warnings and precautions, adverse reactions, drug interactions, use in specific populations, clinical studies
Exemestane	Oncology	ESR, PGR (hormone receptor)	Indications and usage, dosage and administration, clinical studies
Fesoterodine	Urology	CYP2D6	Drug interactions, clinical pharmacology
Flibanserin (1)	Gynecology	CYP2C9	Clinical pharmacology
Flibanserin (2)	Gynecology	CYP2C19	Adverse reactions, use in specific populations, clinical pharmacology
Flibanserin (3)	Gynecology	CYP2D6	Clinical pharmacology
Fluorouracil (1)	Dermatology	DPYD	Contraindications, warnings
Fluorouracil (2)	Oncology	DPYD	Warnings and precautions, patient counseling information
Fluoxetine	Psychiatry	CYP2D6	Precautions, clinical pharmacology
Flurbiprofen	Rheumatology	CYP2C9	Clinical pharmacology
Fluvoxamine	Psychiatry	CYP2D6	Drug interactions
Formoterol (1)	Pulmonary	CYP2D6	Clinical pharmacology
Formoterol (2)	Pulmonary	CYP2C19	Clinical pharmacology
Fulvestrant (1)	Oncology	ERBB2 (HER2)	Indications and usage, adverse reactions, clinical studies
Fulvestrant (2)	Oncology	ESR, PGR (Hormone Receptor)	Indications and usage, adverse reactions, clinical pharmacology, clinical studies
Galantamine	Neurology	CYP2D6	Clinical pharmacology
Gefitinib (1)	Oncology	EGFR	Indications and usage, dosage and administration, clinical studies
Gefitinib (2)	Oncology	CYP2D6	Clinical pharmacology
Glimepiride	Endocrinology	G6PD	Warnings and precautions, adverse reactions
Glipizide	Endocrinology	G6PD	Precautions
Glyburide	Endocrinology	G6PD	Precautions
Hydralazine	Cardiology	Nonspecific (NAT)	Clinical pharmacology
Ibrutinib (1)	Oncology	Chromosome 17p	Indications and usage, clinical studies
Ibrutinib (2)	Oncology	Chromosome 11q	Clinical studies
Iloperidone	Psychiatry	CYP2D6	Dosage and administration, warnings and precautions, drug interactions, clinical pharmacology
Imatinib (1)	Oncology	KIT	Indications and usage, dosage and administration, clinical studies
Imatinib (2)	Oncology	BCR-ABL1 (Philadelphia chromosome)	Indications and usage, dosage and administration, warnings and precautions, adverse reactions, use in specific populations, clinical pharmacology, clinical studies
Imatinib (3)	Oncology	PDGFRB	Indications and usage, dosage and administration, clinical studies
Imatinib (4)	Oncology	FIP1L1-PDGFRA	Indications and usage, dosage and administration, clinical studies
Imipramine	Psychiatry	CYP2D6	Precautions
Indacaterol	Pulmonary	UGT1A1	Clinical pharmacology
Inotuzumab Ozogamicin	Oncology	BCR-ABL1 (Philadelphia chromosome)	Clinical studies
Irinotecan	Oncology	UGT1A1	Dosage and administration, warnings and precautions, clinical pharmacology
Isoflurane	Anesthesiology	Nonspecific (genetic susceptibility to malignant hyperthermia)	Contraindications
Isoniazid, pyrazinamide, and rifampin	Infectious Diseases	Nonspecific (NAT)	Clinical pharmacology
Isosorbide dinitrate	Cardiology	CYB5R	Overdosage
Isosorbide mononitrate	Cardiology	CYB5R	Overdosage
Ivacaftor	Pulmonary	CFTR	Indications and usage, adverse reactions, use in specific populations, clinical pharmacology, clinical studies
Ivacaftor and lumacaftor	Pulmonary	CFTR	Indications and usage, adverse reactions, use in specific populations, clinical studies
Ivacaftor and tezacaftor	Pulmonary	CFTR	Indications and usage, adverse reactions, use in specific populations, clinical pharmacology, clinical studies
Lacosamide	Neurology	CYP2C19	Clinical pharmacology
Lansoprazole	Gastroenterology	CYP2C19	Drug interactions, clinical pharmacology
Lapatinib (1)	Oncology	ERBB2 (HER2)	Indications and usage, dosage and administration, adverse reactions, use in specific populations, clinical studies
Lapatinib (2)	Oncology	ESR, PGR (Hormone Receptor)	Indications and usage, dosage and administration, adverse reactions, use in specific populations, clinical studies
Lapatinib (3)	Oncology	HLA-DQA1, HLA-DRB1	Clinical pharmacology
Ledipasvir and sofosbuvir	Infectious diseases	IFNL3 (IL28B)	Clinical studies
Lenalidomide	Hematology	Chromosome 5q	Boxed warning, indications and usage, adverse reactions, use in specific populations, clinical studies
Lesinurad	Rheumatology	CYP2C9	Drug interactions, clinical pharmacology
Letrozole	Oncology	ESR, PGR (Hormone Receptor)	Indications and usage, adverse reactions, clinical studies
Lidocaine and prilocaine (1)	Anesthesiology	Nonspecific (congenital methemoglobinemia)	Warnings and precautions
Lidocaine and prilocaine (2)	Anesthesiology	G6PD	Warnings and precautions, clinical pharmacology
Lofexidine	Anesthesiology	CYP2D6	Use in specific populations
Mafenide	Infectious diseases	G6PD	Warnings, adverse reactions
Meclizine	Neurology	CYP2D6	Clinical pharmacology
Mercaptopurine (1)	Oncology	TPMT	Dosage and administration, warnings and precautions, adverse reactions, clinical pharmacology
Mercaptopurine (2)	Oncology	NUDT15	Dosage and administration, warnings and precautions, clinical pharmacology
Methylene blue	Hematology	G6PD	Contraindications, warnings and precautions
Metoclopramide (1)	Gastroenterology	CYB5R	Precautions, overdosage
Metoclopramide (2)	Gastroenterology	G6PD	Precautions, overdosage
Metoprolol	Cardiology	CYP2D6	Drug interactions, clinical pharmacology
Midostaurin (1)	Oncology	FLT3	Indications and usage, dosage and administration, adverse reactions, clinical studies
Midostaurin (2)	Oncology	NPM1	Clinical studies
Midostaurin (3)	Oncology	KIT	Clinical studies
Mirabegron	Urology	CYP2D6	Clinical pharmacology
Modafinil	Psychiatry	CYP2D6	Clinical pharmacology
Mycophenolic acid	Transplantation	HPRT1	Warnings and precautions
Nalidixic acid	Infectious diseases	G6PD	Precautions, adverse reactions
Nebivolol	Cardiology	CYP2D6	Dosage and administration, clinical pharmacology
Nefazodone	Psychiatry	CYP2D6	Precautions
Neratinib (1)	Oncology	ERBB2 (HER2)	Indications and usage, adverse reactions, clinical studies
Neratinib (2)	Oncology	ESR, PGR (Hormone Receptor)	Clinical studies
Nilotinib (1)	Oncology	BCR-ABL1 (Philadelphia chromosome)	Indications and usage, dosage and administration, warnings and precautions, adverse reactions, use in specific populations, clinical studies
Nilotinib (2)	Oncology	UGT1A1	Clinical pharmacology
Niraparib	Oncology	BRCA	Clinical studies
Nitrofurantoin	Infectious Diseases	G6PD	Warnings, adverse reactions
Nivolumab (1)	Oncology	BRAF	Indications and usage, adverse reactions, clinical studies
Nivolumab (2)	Oncology	CD274 (PD-L1)	Clinical pharmacology, clinical studies
Nivolumab (3)	Oncology	Microsatellite instability, mismatch repair	Indications and usage, use in specific populations, clinical pharmacology, clinical studies
Nortriptyline	Psychiatry	CYP2D6	Precautions
Obinutuzumab	Oncology	MS4A1 (CD20 antigen)	Clinical studies
Olaparib	Oncology	BRCA	Indications and usage, dosage and administration, warnings and precautions, adverse reactions, clinical studies
Olaratumab	Oncology	PDGFRA	Clinical studies
Omacetaxine	Oncology	BCR-ABL1 (Philadelphia chromosome)	Clinical studies
Ombitasvir, paritaprevir, and ritonavir	Infectious diseases	IFNL3 (IL28B)	Clinical studies
Omeprazole	Gastroenterology	CYP2C19	Drug interactions, clinical pharmacology
Ondansetron	Gastroenterology	CYP2D6	Clinical pharmacology
Osimertinib	Oncology	EGFR	Indications and usage, dosage and administration, adverse reactions, clinical studies
Oxcarbazepine	Neurology	HLA-B	Warnings and precautions
Palbociclib (1)	Oncology	ESR (Hormone Receptor)	Indications and usage, adverse reactions, clinical studies
Palbociclib (2)	Oncology	ERBB2 (HER2)	Indications and usage, adverse reactions, clinical studies
Palonosetron	Gastroenterology	CYP2D6	Clinical pharmacology
Panitumumab (1)	Oncology	EGFR	Adverse reactions, clinical pharmacology, clinical studies
Panitumumab (2)	Oncology	RAS	Indications and usage, dosage and administration, warnings and precautions, adverse reactions, clinical studies
Pantoprazole	Gastroenterology	CYP2C19	Clinical pharmacology
Parathyroid hormone	Inborn errors of metabolism	CASR	Indications and usage, clinical studies
Paroxetine	Psychiatry	CYP2D6	Drug interactions
Pazopanib (1)	Oncology	UGT1A1	Clinical pharmacology
Pazopanib (2)	Oncology	HLA-B	Clinical pharmacology
Peginterferon alfa-2b	Infectious Diseases	IFNL3 (IL28B)	Clinical pharmacology
Pegloticase	Rheumatology	G6PD	Boxed warning, contraindications, warnings and precautions, patient counseling information
Pembrolizumab (1)	Oncology	BRAF	Adverse reactions, clinical studies
Pembrolizumab (2)	Oncology	CD274 (PD-L1)	Indications and usage, dosage and administration, use in specific populations, clinical studies
Pembrolizumab (3)	Oncology	Microsatellite Instability, Mismatch Repair	Indications and usage, Dosage and administration, use in specific populations, clinical studies
Perphenazine	Psychiatry	CYP2D6	Precautions, clinical pharmacology
Pertuzumab (1)	Oncology	ERBB2 (HER2)	Indications and usage, warnings and precautions, adverse reactions, clinical pharmacology, clinical studies
Pertuzumab (2)	Oncology	ESR, PGR (Hormone Receptor)	Clinical studies
Phenytoin (1)	Neurology	CYP2C9	Clinical pharmacology
Phenytoin (2)	Neurology	CYP2C19	Clinical pharmacology
Phenytoin (3)	Neurology	HLA-B	Warnings
Pimozide	Psychiatry	CYP2D6	Dosage and administration, precautions
Piroxicam	Rheumatology	CYP2C9	Clinical pharmacology
Ponatinib	Oncology	BCR-ABL1 (Philadelphia chromosome)	Indications and usage, warnings and precautions, adverse reactions, use in specific populations, clinical studies
Prasugrel (1)	Cardiology	CYP2C19	Use in specific populations, clinical pharmacology, clinical studies
Prasugrel (2)	Cardiology	CYP2C9	Use in specific populations, clinical pharmacology, clinical studies
Prasugrel (3)	Cardiology	CYP3A5	Use in specific populations, clinical pharmacology, clinical studies
Prasugrel (4)	Cardiology	CYP2B6	Use in specific populations, clinical pharmacology, clinical studies
Primaquine (1)	Infectious Diseases	G6PD	Contraindications, warnings, precautions, adverse reactions, overdosage
Primaquine (2)	Infectious Diseases	CYB5R	Precautions, adverse reactions
Propafenone	Cardiology	CYP2D6	Dosage and administration, warnings and precautions, drug interactions, clinical pharmacology
Propranolol	Cardiology	CYP2D6	Clinical pharmacology
Protriptyline	Psychiatry	CYP2D6	Precautions
Quinidine	Cardiology	CYP2D6	Precautions
Quinine sulfate (1)	Infectious Diseases	G6PD	Contraindications
Quinine sulfate (2)	Infectious Diseases	CYP2D6	Drug interactions
Rabeprazole	Gastroenterology	CYP2C19	Clinical pharmacology
Raltegravir	Infectious Diseases	UGT1A1	Clinical pharmacology
Rasburicase (1)	Oncology	G6PD	Boxed warning, contraindications, warnings and precautions
Rasburicase (2)	Oncology	CYB5R	Boxed warning, contraindications, warnings and precautions
Ribociclib (1)	Oncology	ESR, PGR (Hormone Receptor)	Indications and usage, clinical studies
Ribociclib (2)	Oncology	ERBB2 (HER2)	Indications and usage, clinical studies
Risperidone	Psychiatry	CYP2D6	Drug interactions, clinical pharmacology
Rituximab	Oncology	MS4A1 (CD20 antigen)	Indications and usage, dosage and administration, adverse reactions, use in specific populations, clinical studies
Rosuvastatin	Endocrinology	SLCO1B1	Clinical pharmacology
Rucaparib (1)	Oncology	BRCA	Indications and usage, dosage and administration, adverse reactions, use in specific populations, clinical studies
Rucaparib (2)	Oncology	CYP2D6	Clinical pharmacology
Rucaparib (3)	Oncology	CYP1A2	Clinical pharmacology
Sevoflurane	Anesthesiology	Nonspecific (Genetic Susceptibility to Malignant Hyperthermia)	Warnings
Simeprevir	Infectious Diseases	IFNL3 (IL28B)	Clinical pharmacology, clinical studies
Sodium nitrite	Toxicology	G6PD	Warnings and precautions
Sofosbuvir	Infectious Diseases	IFNL3 (IL28B)	Clinical studies
Sofosbuvir and velpatasvir	Infectious Diseases	IFNL3 (IL28B)	Clinical studies
Sofosbuvir, velpatasvir, and voxilaprevir	Infectious Diseases	IFNL3 (IL28B)	Clinical studies
Succimer	Hematology	G6PD	Clinical pharmacology
Succinylcholine	Anesthesiology	BCHE	Warnings, precautions
Sulfamethoxazole and trimethoprim (1)	Infectious Diseases	G6PD	Precautions
Sulfamethoxazole and trimethoprim (2)	Infectious Diseases	Nonspecific (NAT)	Precautions
Sulfasalazine (1)	Gastroenterology	G6PD	Precautions
Sulfasalazine (2)	Gastroenterology	Nonspecific (NAT)	Clinical pharmacology
Tamoxifen (1)	Oncology	ESR, PGR (Hormone receptor)	Indications and usage, precautions, adverse reactions, clinical studies
Tamoxifen (2)	Oncology	F5 (Factor V Leiden)	Warnings
Tamoxifen (3)	Oncology	F2 (Prothrombin)	Warnings
Telaprevir	Infectious Diseases	IFNL3 (IL28B)	Clinical pharmacology, clinical studies
Tetrabenazine	Neurology	CYP2D6	Dosage and administration, warnings and precautions, use in specific populations, clinical pharmacology
Thioguanine (1)	Oncology	TPMT	Dosage and administration, warnings, precautions, clinical pharmacology
Thioguanine (2)	Oncology	NUDT15	Dosage and administration, warnings, precautions, clinical pharmacology
Thioridazine	Psychiatry	CYP2D6	Contraindications, warnings, precautions
Ticagrelor	Cardiology	CYP2C19	Clinical pharmacology
Tolterodine	Urology	CYP2D6	Precautions, clinical pharmacology
Tramadol	Anesthesiology	CYP2D6	Boxed warning, warnings, precautions, use in specific populations, clinical pharmacology
Trametinib (1)	Oncology	BRAF	Indications and usage, dosage and administration, adverse reactions, clinical pharmacology, clinical studies, patient counseling information
Trametinib (2)	Oncology	G6PD	Adverse reactions
Trametinib (3)	Oncology	RAS	Warnings and precautions
Trastuzumab (1)	Oncology	ERBB2 (HER2)	Indications and usage, warnings and precautions, clinical pharmacology, clinical studies
Trastuzumab (2)	Oncology	ESR, PGR (Hormone receptor)	Clinical studies
Tretinoin	Oncology	PML-RARA	Indications and usage, warnings, clinical pharmacology
Trimipramine	Psychiatry	CYP2D6	Precautions
Umeclidinium	Pulmonary	CYP2D6	Clinical pharmacology
Ustekinumab	Dermatology and gastroenterology	IL12A, IL12B, IL23A	Warnings and precautions
Valbenazine	Neurology	CYP2D6	Dosage and administration, warnings and precautions, use in specific populations, clinical pharmacology
Valproic acid (1)	Neurology	POLG	Boxed warning, contraindications, warnings and precautions
Valproic acid (2)	Neurology	Nonspecific (urea cycle disorders)	Contraindications, warnings and precautions
Vemurafenib (1)	Oncology	BRAF	Indications and usage, dosage and administration, warnings and precautions, adverse reactions, use in specific populations, clinical pharmacology, clinical studies, patient counseling information
Vemurafenib (2)	Oncology	RAS	Warnings and precautions, adverse reactions
Venlafaxine	Psychiatry	CYP2D6	Precautions
Venetoclax	Oncology	Chromosome 17p	Indications and usage, dosage and administration, use in specific populations, clinical studies
Voriconazole	Infectious Diseases	CYP2C19	Clinical pharmacology
Vortioxetine	Psychiatry	CYP2D6	Dosage and administration, clinical pharmacology
Warfarin (1)	Hematology	CYP2C9	Dosage and administration, drug interactions, clinical pharmacology
Warfarin (2)	Hematology	VKORC1	Dosage and administration, clinical pharmacology
Warfarin (3)	Hematology	PROS1	Warnings and precautions
Warfarin (4)	Hematology	PROC	Warnings and precautions

The table lists 355 BMs as of December 2018, linked to drugs with pharmacogenomic information found in the drug labeling. The list of BMs includes but are not limited to germline or somatic gene variants (i.e. polymorphisms, mutations), functional deficiencies with a genetic etiology, altered gene expression signatures, and chromosomal abnormalities, and selected protein BMs that are used to select treatments for patients (adapted from Drugs@FDA; https://www.fda.gov/drugs/scienceresearch/ucm572698.htm)

Moreover, as reported recently by Burke [10] there are more than 768,000 papers indexed in PubMed.gov directly related to BMs (https://www.amplion.com/biomarker-trends/biomarker-panels-the-good-the-bad-and-the-ugly/).

All the data collected so far have shown insufficient linkages between BMs and disease pathogenesis resulting in the failure of many BMs as well as drug targets. It is critical to link the target to the disease pathogenesis thus, enabling the development of better and more precise therapies by pre-selecting responders to treatment.

### Biomarkers and decision making

BMs have been used to improve patient’s stratification and/or develop targeted therapies facilitating the decision-making process throughout the new drug development process. BMs constitute a rational approach which, at its most optimal, reflects both the biology of the disease and the effectiveness of the drug candidate. Also, adding the appropriate BMs to a drug-development strategy enables the concept of ‘fail fast, fail early’; thus, allowing early identification of the high proportion of compounds that fail during drug development. Reducing human exposure to drugs with low efficacy or safety concerns allows to shift resources to drugs that have a higher chance of success. Identification of BMs helpful for a quick go-no-go decision early in the drug development process is critical for enhancing the probability of success of a drug.

Traditionally, clinical trial end-points, such as morbidity and mortality, often require extended timeframes and may be difficult to evaluate. Imaging-based BMs are providing objective end-points that may be confidently evaluated in a reasonable timeframe. However, imaging techniques are rather expensive and often very impractical especially in specific geographical area.

Despite all of these, BMs are essential for deciding which patients should receive a specific treatment. Table 1 illustrates a number or pharmacogenomic BMs in drug labeling. As of December 2018, approximately 355 pharmacogenomic BMs are linked to drugs with pharmacogenomic information found in the drug labeling. These BMs include germline or somatic gene variants (i.e. polymorphisms, mutations), functional deficiencies with a genetic etiology, altered gene expression signatures, and chromosomal abnormalities, and selected protein BMs that are used to select treatments for patients.

Pre-clinical BMs are essential, as long they translate into clinical markers. Which is often is not the case. Several reasons can be offered to explain why many clinical studies have failed to identify BMs ability to predict treatment efficacy or disease modification including lack of statistical power, lack of validation standards [11] and pharmacogenetic heterogeneity of patient groups [12].

### Genomics, epigenetics, and microRNAs as emerging biomarkers in cancer, diabetes, autoimmune and inflammatory diseases

Biomarkers with the potential to identify early stages of disease for example pre-neoplastic disease or very early stages of cancer are of great promise to improve patient survival. The concept of liquid biopsy refers to a minimally invasive collection and analysis of molecules that can be isolated from body fluids, primarily whole blood, serum, plasma, urine and saliva, and others. A myriad of circulating molecules such as cell-free DNA (cf-DNA), cell-free RNA (cf-RNA) including microRNAs (miRNAs), circulating tumor cells (CTC), circulating tumor proteins, and extracellular vesicles, more specifically exosomes, have been explored as biomarkers [13].

Genetic and epigenetic alterations including DNA methylation and altered miRNA expression might be contributing to several autoimmune diseases, cancer, transplantation, and infectious diseases. For example in a recent study in rheumatoid arthritis (RA), de la Rica et al. [14] has identified epigenetic factors involved in RA, and hence conducted DNA methylation and miRNA expression profiling of a set of RA synovial fibroblasts and compared the results with those obtained from osteoarthritis (OA) patients with a normal phenotype. In this study, researchers identified changes in novel key genes including IL6R, CAPN8, and DPP4, as well as several HOX genes. Notably, many genes modified by DNA methylation were inversely correlated with expression miRNAs. A comprehensive analysis revealed several miRNAs that are controlled by DNA methylation, and genes that are regulated by DNA methylation and targeted by miRNAs were of potential use as clinical markers. The study found that several genes including Stat4 and TRAF1-C5 were identified as risk factors contributing to RA and other autoimmune diseases such as SLE [15, 16]. RA is also strongly associated with the inherited tissue type MHC antigen HLA-DR4 and the genes PTPN22 and PAD14 [15]. DNA methylation screening identified genes undergoing DNA methylation-mediated silencing including IL6R, CAPN8 and DPP4, as well as several HOX genes; and a panel of miRNAs that are controlled by DNA methylation, and genes that are regulated by DNA methylation and are targeted by miRNAs.

Likewise, changes in miRNA levels in blood and other body fluids (miRNAs) have been linked to a variety of autoimmune diseases [17] including: (i) Type 1 diabetes, miR-342, miR-191, miR-375 and miR-21 and miR-510 and others [18–20]; (ii) Type 2 diabetes, miR-30, miR-34a, miR-145 and miR-29c, miR-138, -192, -195, -320b, and let-7a, (iii) prediabetes (miR-7, miR-152 and miR-192) [21, 22] and insulin resistance (miR-24, miR-30d, miR-146a), obesity and metabolic diseases [19–26] (iv) Multiple sclerosis (MS), miR-326 [27], miR-17-5p [28]; (v) Rheumatoid Arthritis (RA), miR-146a, miR-155 and miR-16 [29, 30]; (vi) Primary biliary cirrhosis, miR-122a, miR-26a, miR-328, miR-299-5p [31]; (vii) Sjögren’s syndrome, miR-17-92 [17]; (viii) SLE, miR-146a [32], miR-516-5p, miR-637 [33]; and (ix) Psoriasis, miR-203, miR-146a, miR125b, miR21 [34].

In the case of RA, alterations in several miRNAs expression patterns including miR-146a, miRNA-155, miRNA-124a, miR-203, miR-223, miR-346, miR-132, miR-363, miR-498, miR-15a, and miR-16 were documented in several tissue samples of RA patients. The polymorphisms present in these miRNAs and their targets have also been associated with RA or other autoimmune diseases [19, 35]. Several reports have shown altered miRNA expression in the synovium of patients with RA [36]. For example, elevated expression of miR-346 was found in Lipopolysaccharide activated RA fibroblast-like synoviocytes (FLS) [37]. Moreover, miR-124 was found at lower levels in RA FLS in comparison with FLS from patients with OA [38]. miR-146a has been found to be elevated in human RA synovial tissue and its expression is induced by the pro-inflammatory cytokines i.e. tumor necrosis factor and interleukin1β [29]. Furthermore, miR-146, miR-155, and miR-16 were all elevated in the peripheral blood of RA patients with the active disease rather than inactive disease [30] suggesting that these miRNAs may serve as potential disease activity markers.

The epigenetic regulation of DNA processes has been extensively studied over the past 15 years in cancer, where DNA methylation and histone modification, nucleosome remodeling and RNA mediated targeting regulate many biological processes that are crucial to the genesis of cancer. The first evidence indicating of an epigenetic link with cancer were studied derived from DNA methylation. Though many of the initial studies were purely correlative, however, they did highlight a potential connection between epigenetic pathways and cancer. These preliminary results were confirmed by recent results from the International Cancer Genome Consortium (ICGC).

Compilation of the epigenetic regulators mutated in cancer highlights histone acetylation and methylation as the most widely affected epigenetic pathways. Deep sequencing technologies aimed at mapping chromatin modifications have begun to shed some lights on the origin of epigenetic abnormalities in cancer. Several pieces of evidence are now highlighting that dysregulation of the epigenetic pathways can lead to cancer. All the evidence collected thus far along with clinical and preclinical results observed with epigenetic drugs against chromatin regulators, point to the necessity of embracing a central role of epigenetics in cancer. Unfortunately, those studies are far too many to be comprehensively described in this review.

Furthermore, cancer cell lines have been used to identify potential novel biomarkers for drug resistance and novel targets and pathways for drug repurposing. For example, previously we conducted a functional shRNA screen combined with a lethal dose of neratinib to discover chemo-resistant interactions with neratinib. We identified a collection of genes whose inhibition by RNAi led to neratinib resistance including genes involved in oncogenesis, transcription factors, cellular ion transport, protein ubiquitination, cell cycle, and genes known to interact with breast cancer-associated genes [39]. These novel mediators of cellular resistance to neratinib could lead to their use as patient or treatment selection biomarkers.

In addition, we undertook a genome-wide pooled lentiviral shRNA screen to identify synthetic lethal or enhancer (synthetic modulator screen) genes that interact with sub-effective doses of neratinib in a human breast cancer cell line. We discovered a diverse set of genes whose depletion selectively impaired or enhanced the viability of cancer cells in the presence of neratinib. Further examination of these genes and pathways led to a rationale for the treatment of cells with either paclitaxel or cytarabine in combination with neratinib which resulted in a strong antiproliferative effect. Notably, our findings support a paclitaxel and neratinib phase II clinical trial in breast cancer patients [40].

### Biomarker multiplexing

Multiple biomarkers are used to empower more accurate patient stratification. To improve patient stratification for immunotherapy, the analysis of immuno-oncology biomarkers, like PD-L1, as well as a more comprehensive analysis of the immune and tumor-related pathways (the “Cancer Immunogram) (Fig. 2) [4] has to be used for a better patient stratification in future immunotherapy trials [5]. The “Cancer Immunogram” includes tumor foreignness, immune status, immune cell infiltration, absence of checkpoints, absence of soluble inhibitors, absence of inhibitory tumor metabolism, and tumor sensitivity to immune effectors as the most important predictors of immunotherapy response in a single tissue sample [5]. As depicted in Fig. 2, The “Cancer Immunogram” integrates both tumor- and immune-related characteristics assessed with both molecular and image-based methods for individualized prediction of immunotherapy response. By evaluating dynamic data on tissue-based parameters, (*e.g.*, immune cell infiltration and expression of immune checkpoints), quantitative pathology methods are ideally suited for data integration with molecular parameters.

**Fig. 3:**
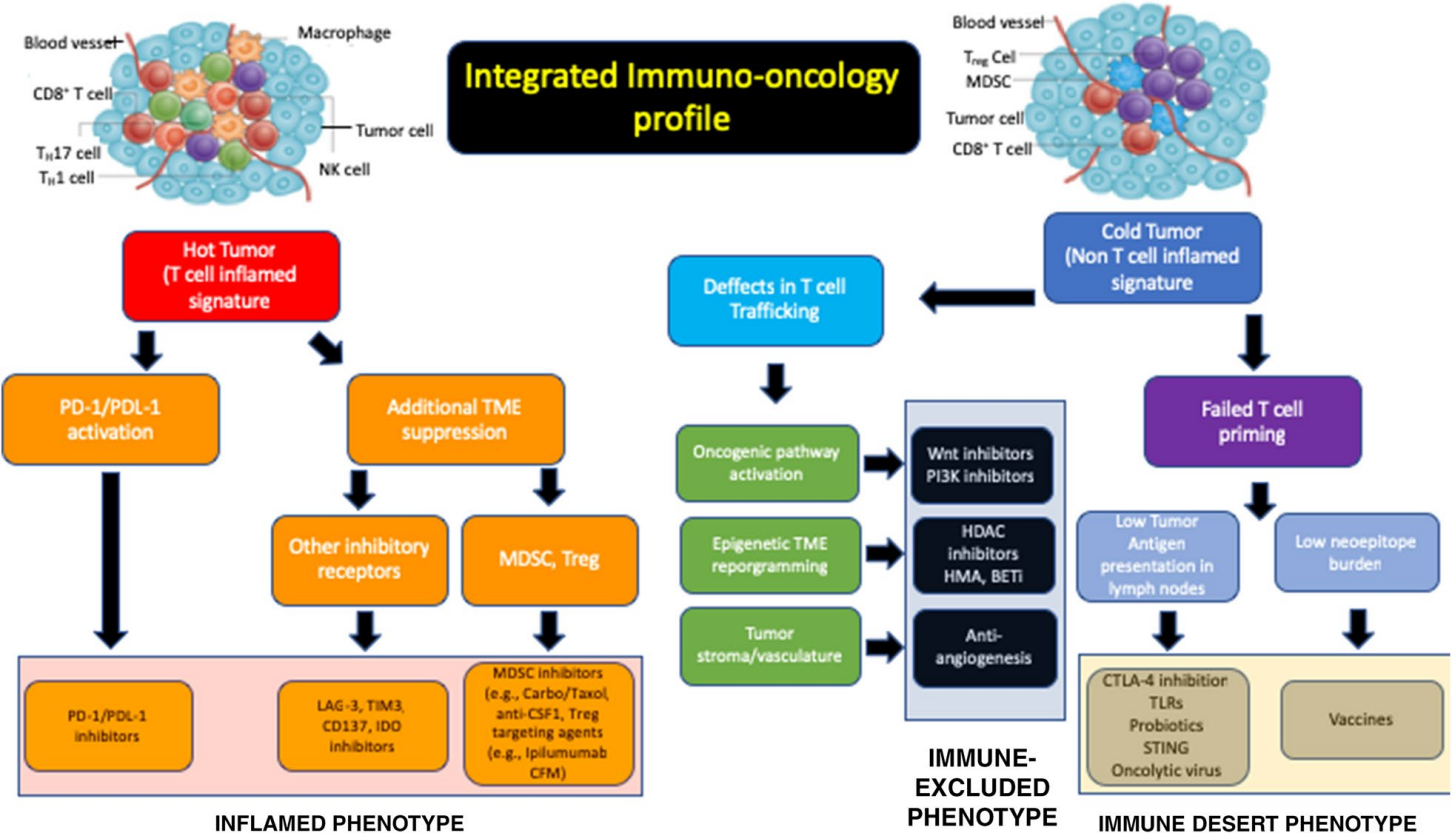
Schematic of an integrated biologic information for a targeted therapeutic intervention. Ag, antigen; BETi, inhibitors of bromodomain and extraterminal proteins; carbo, carboplatin; CSF1, colony stimulating factor 1; CFM, cyclophosphamide; CTLA-4, cytotoxic T-lymphocyte-associated antigen 4; HDAC, histone deacetylase; HMA, hypomethylating agents; IDO, indoleamine 2,3-dioxyenase; IO, immune-oncology; LN, lymph nodes; LAG-3, lymphocyte-activation gene 3; MDSC, myeloid-derived suppressor cells; P13K, phosphoinositide 3-kinase; PD-1, programmed cell death-1; PD-L1, programmed cell death-ligand 1; STING, stimulator of interferon genes; TIM3, T cell immunoglobulin and mucin domain 3; TME, tumor microenvironment; Treg, regulatory T cells; TLR, toll-like receptor; Wnt, wingless, int-1 (adapted from [3, 42])

As illustrated in Fig. 3, and reported in a recent article [3], the utility of this approach to organize and integrate the biologic information into a useful and informative single assay able to inform and influence drug development, personalized therapy strategy and selection of specific patient populations. The authors [3] suggest that anti-cancer immunity can be histologically segregated into three main phenotypes: (1) the inflamed phenotype (“hot” tumors); (2) the immune-excluded phenotype (“cold” tumors); and (3) the immune-desert phenotype (“cold” tumors) [41, 42] (Fig. 3). Each tumor phenotype is associated with specific underlying biological and pathological mechanisms that may determine the success of the host immune response and immunotherapy or other therapeutic modalities to fight cancer. Identifying these mechanisms at the level of the individual groups of patients and selecting those patients with similar tumor phenotype is critical for the selection of specific patient populations both for the development as well as implementation of therapeutic interventions.

### Digital biomarkers

Digital BMs are defined as an objective, quantifiable physiological and behavioral data that are collected and measured by means of digital devices. The data collected is typically used to explain, influence and/or predict health-related outcomes. Increasingly, many smartphone apps are also available for health management with or without connection to these sensor devices [43, 44]. There are approx. 300,000 health apps and 340 + (CK personal communication) sensor devices available today and the number of apps is doubling every 2 years. Recently, a new class of wearable smartphone-coupled devices such as smart watches have been widely available. These devices offer new, and more practical opportunities not without limitations [44]. As those wearable devices and their corresponding apps continue to develop and evolve, there will be a need for a more dedicated research and digital expert assessment to evaluate different healthcare applications as well as assess the limitations and the risks of impinging on the individual privacy and data safety.

This surge in technology has made it possible for ‘consumers’ to track their health but also represents an interesting opportunity to monitor healthcare and clinical trials. Data collected about a patient’s activity and vital signs can be used to get an idea about the patient’s health status and disease progression on a daily basis. However, the problem is that a majority of these apps and devices are meant for wellness purposes and not intended to diagnose or treat diseases.

As reported previously in the literature [5], and shown Figs. 1 and 2, recent advances in electronic data collection will be instrumental in our ability to digitize and process large collections of tissue slides and molecular diagnostic profiling. The evolving field of machine learning and artificial intelligence with the support of human interpretation will have a dramatic impact on the field [45, 46].

This field has already generated tangible results. Indeed,, medical device companies (e.g., Philips, GE, and Leica) are developing new imaging technologies for digital pathology to detect digital biomarkers, while a number of Information Technology (IT) companies (e.g., Google, IBM, and Microsoft, or PathAI) are developing tools, such as machine learning and artificial intelligence (AI) for big data analysis and integrated decision making.

Pharmaceutical companies are also moving in the same direction. For example, FDA clearance for the VENTANA MMR IHC Panel for patients diagnosed with colorectal cancer (CRC) developed by Roche is a demonstration of these efforts [5]. Thus, developing digital biomarkers, big data analysis and interpretation will be beneficial in the new era of PM.

#### How can wearable help in clinical trials and healthcare?

In a typical clinical trial or in a clinical setting, the patient visits the hospital not more than once per month or less. So, the clinician can observe the signs and symptoms of the patient only during this visit and has almost no visibility on how the patient is doing for the majority of the time outside the clinic. If digital BMs are used, the patient can perform these tests using smartphones or sensors in the comfort of his/her home. For example, in a Parkinson’s disease trial various aspects of the patient’s health can be captured in a remote study using smartphone-based apps. This allows the collection of quantitative and unbiased data on a frequent or almost continuous basis. The clinician can get almost real-time feedback on each patient, whether they are getting better or worse. This feedback can help to inform the study protocol or even halt the study if the drug doesn’t seem to be working on most of the patients.

The Clinical Trials Transformation Initiative (CTTI) provides a framework and detailed guidance for developing digital BMs. They also outline the benefits of using digital BMs in clinical trials such as being patient-centric while also making faster decisions that save time and costs.

#### Develop and validate digital biomarkers

The first and most important consideration in developing digital BMs is not which device to use, but rather deciding which disease symptoms to capture that best represent the disease. Involving patients, and physicians in the discussion are necessary to understand which symptoms matter to patients. At the same time, it is important to consider if these symptoms can be objectively measured and what is a meaningful change in measurement that reflects treatment benefit.

Once it is clear what endpoints need to be captured, the right device can be selected. The device technology needs to be verified (measurement errors, variances, etc.) and the device also needs to be validated for the specific use (reliability; accuracy and precision compared to gold standard or independent measurements). An observational study is required to ensure the suitability of the device before deploying it in a trial.

#### Diseases that can be tracked with digital biomarkers

Heart disease and diabetes measurements are common application areas for sensor-based devices. However, digital BMs could have the most impact in monitoring CNS diseases since it gives us the opportunity to measure symptoms that were largely intractable until now. Various sensor devices are available for tracking several aspects of health such as activity, heart rate, blood glucose and even sleep, breath, voice, and temperature. Most smartphones are equipped with several sensors that can perform the various motion, sound and light based tests. In addition, the smartphone can be used for psychological tests or to detect finger motions through the touchscreen. These measures can be used in various combinations to predict the health aspects or symptoms required.

Digital BMs can have several applications beyond clinical trials, for example in diagnostics—to identify patients affected by a disease. However, the most interesting application is in digital therapeutics where the device/app can be used to help the treatment like insulin dose adjustment or to monitor/treat substance abuse or addiction. Digital BMs present a big opportunity for measuring endpoints in a remote, objective and unbiased manner that was largely difficult until now. However, there are still several challenges that need to be considered before developing and deploying them to measure endpoints in clinical trials.

### The conundrum of biomarker strategy

There is a wrong notion that by the time a BM is discovered and validated; it is too late to affect the decision-making process. The real question is whether the chosen BM is: (1) intrinsically related to the pathogenesis of a disease; and (2) whether it is reliable and adequate for decision-making. It has been reported that building computer models can transform potential BM into clinically meaningful tests. However, on several occasions when scientists [47] attempted to import data from the literature, they found that the diagnostic criteria used to assess BMs accuracy were vague or based on un-validated BMs.

Identifying BMs that can be translated from animal models to humans is also challenging [48]. While inhibiting an enzyme in an animal model may be effective, this may not be the case in humans. This is either because the pathway has diverged or humans have some compensatory mechanisms. A treatment may change a BM, but this may be irrelevant to a specific disease. Therefore, a true BM must be intrinsically linked to the pathogenesis of the disease. A drug should treat a disease, not a BM.

Without understanding the pathogenesis of a disease, it is difficult to figure out what is the right BM to be used in clinical studies. Once a BM is identified, it is difficult to understand whether it is associated with a specific disease or multiple diseases or if it is a reflection of poor health. For instance, if you are studying potential BMs for Systemic Lupus Erythematosus (SLE) or Alzheimer’s Disease (AD), the same set of BMs keeps emerging as potential differentiators. A growing body of evidence indicates that SLE is associated with increased risk of cognitive impairment and dementia [49]. The real question is, however, whether those specific BMs would be able to differentiate SLE from AD. Otherwise, the plethora of BMs that has been generated would be irrelevant.

Pharmaceutical companies are obsessed with the idea that a BM needs to be validated before it can be used for decision-making. Unfortunately, there are no clear-cut criteria to date identifying which BM should be validated. The rigor on how to use a BM to kill a compound relies entirely on the discretion of pharmaceutical companies. The risk of using the wrong BM or selecting the wrong set of BMs may lead to the wrong decision of dumping a good drug because the adopted BM strategy was evaluated inaccurately. To overcome this problem, pharmaceutical companies tend to rely their decision-making process on a long list of BMs (very often too many). This is based on the notion that clusters of variables can be used to differentiate responders from non-responders. The risk of utilizing a long list of BMs is not only costly but also to make the data difficult to be interpreted. The best solution to this problem is to select a strategy that selects a few BMs with complementary predictive properties.

In the last few years, the FDA has pressured pharmaceuticals to shift the paradigm towards PM, thus targeting diagnostics and treatments based on patient-stratification. This has prompted everyone in the pharmaceutical field to translate molecular profiles into effective treatments, thus impacting: (i) prevention; (ii) early detection; (iii) use of animal or in silico models to facilitate the prediction of success by increasing efficacy and minimizing toxicity and (iv) computational biology to create new synergies between discovery and drug development.

### Computational biology and bioinformatics to aid biomarker development

There is a need to develop novel computer-aided algorithms and methodologies for pattern recognition, visualization, and classification of distribution metrics for interpreting large sets of data coming from high-throughput molecular profiling studies. This is where the bioinformatics and computational biology play a critical role in linking biological knowledge with clinical practice: they are the interface between the clinical development process of drug target and BM discovery and development.

Computational biology uses computational tools and machine learning for data mining, whereas bioinformatics applies computing and mathematics to the analysis of biological datasets to support the solution of biological problems. Bioinformatics plays a key role in analyzing data generated from different ‘omics’ platforms annotating and classifying genes/pathways for target identification and disease association.

The goal of bioinformaticians is to use computational methods to predict factors (genes and their products) using: (1) a combination of mathematical modeling and search techniques; (2) mathematical modeling to match and analyze high-level functions; and (3) computational search and alignment techniques to compare new biomolecules (DNA, RNA, protein, metabolite, etc.) within each functional ‘omics’ platform. Combination of this and patient datasets are then used to generate hypotheses.

Bioinformatics and computational biology enable fine tuning of hypotheses [50]. These fields often require specialized tools and skills for data exploration, clustering, regression and supervised classification [51, 52], pattern recognition and selection [53], and development of statistical filtering or modeling strategies and classifiers including neural networks or support vector machines.

The integration of clinical and ‘omics’ data sets has allowed the exploitation of available biological data such as functional annotations and pathway data [54–56]. Consequently, this has led to the generation of prediction models of disease occurrence or responses to therapeutic intervention [51, 57].

However, the use of high throughput “omics” technologies and large sample sizes have generated massive amounts of data sets and their analyses have become a major bottleneck requiring sophisticated computational and statistical methods and skill sets to analyze them [9].

### The role of modeling and simulation to support information-based medicine

Modeling and simulation (M&S) can accelerate drug development and reduce costs significantly [58]. It relies on a feedback loop leading to the production of more relevant compounds to feed into the development cycle. M&S begins with a new data set, such as BMs to link bench to bedside, thus generating a feedback loop with the drug development cycle. Once the right data is available, investigators can test hypotheses to understand the molecular factors contributing to disease and devising better therapies and simulating different study designs before testing the drug candidate in a clinical trial.

The utility of this approach was shown by Roche AG receiving approval for a combination drug (PEGASYS) for the treatment of hepatitis C. The approach used a variety of factors, including the genotype of the virus and the weight of the patient to select the proper dose for a subset of patients. Pfizer was also pioneering this approach for Neurontin (gabapentin). This drug was approved for a variety of neuropathic pain disorders, including post-herpetic neuralgia. Despite those examples, many companies have not yet fully embraced this approach and are still struggling with modeling and simulation tools, due to poor integration of separate data sets. The tools developed for data integration do not communicate well with each other since they rely on data that are in separate databases. Based on this, it will be difficult to include M&S as an integral part of the development process, unless companies integrate their systems more seamlessly. All the odds speak in favor of the fact that industries are adopting standard data formats and managing structured (data in databases) and unstructured data (documents) sets. As a result, the outcome of translating drug development into clinical practice will be more efficient.

Using pharmacogenomic data, M&S can help us to unravel critical safety issues. FDA has started to recognize with the Critical Path initiative the value of M&S as an important part of the CRADA in 2006 (US Food and Drug Administration, “Challenge and Opportunity on the Critical Path to New Medical Products”).

The goal of CRADA is to develop software to support CDISC data formats that can link to other FDA databases and which can ultimately conduct modeling and simulation. This data will ultimately be applied to the end of Phase IIa revision to make a go or no-go decision.

### Machine learning and artificial intelligence can improve precision medicine

The recent big data revolution, accompanied with the generation of continuously collected large data set from various molecular profiling (genetic, genomic, proteomic, epigenomic and others) efforts of patient samples by the development and deployment of wearable medical devices (e.g. wearable watches) and mobile health applications, and clinical outcome data has enabled the biomedical community to apply artificial intelligence (AI) and machine learning algorithms to vast amounts of data. These technological advancements have created new research opportunities in predictive diagnostics, precision medicine, virtual diagnosis, patient monitoring, and drug discovery and delivery for targeted therapies. These advancements have awoken the interests of academic, industry researchers, and regulatory agencies alike and are already providing new tools to physicians.

An example is the application of precision immunoprofiling by image analysis and artificial intelligence to biology and disease. This was demonstrated in a recent paper where the authors used immunoprofiling data to assess immuno-oncology biomarkers, such as PD-L1 and immune cell infiltrates as predictors of patient’s response to cancer treatment [5]. Through spatial analysis of tumor-immune cell interactions, multiplexing technologies, machine learning, and AI tools these authors demonstrated the utility of pattern-recognition in large and complex datasets and deep learning approaches for survival analysis [5].

Essentially, we are using genetics, epigenetics, genomics, proteomics, and other molecular profiling data to inform biology, which we then are evaluating progressively backward using clinical, cellular, and in vitro assays for the discovery of novel targets, pathways, and BMs. Using this plethora of data and data on drugs, we are in a position to come up with candidate drugs faster that most likely work as compared to rational drug design. The goal for human exploratory data would be to aggregate data across the entire medical ecosystem, and give it to third parties to analyze. The pharmaceutical industry could then use AI to build models or to surface patterns—connecting with the patient outcome data—to provide insights into potential benefits to patients. To accomplish this, it is going to take academia, government, and industry—society at large to make better use of human exploratory data. Up to date, the only way to streamline access to human exploratory data is if patients consent, so part of the solution is patient empowerment.

A recent publication [59] highlights the potential utility of AI in cancer diagnostics. Scientists re-trained an off-the-shelf Google deep learning algorithm to identify the most common types of lung cancers with 97% accuracy that even identified altered genes driving abnormal cell growth. To accomplish this, scientists fed Inception v3 slide images supplied by The Cancer Genome Atlas, a database consisting of images of cancer histopathology data and the associated diagnostic annotations. This type of AI has been used to identify faces, animals, and objects in pictures uploaded to servers portal (i.e. Google’s online services) has proven useful at diagnosing the disease before, including diabetic blindness and heart conditions. The researchers found the AI performed almost as well as experienced pathologists when it was used to distinguish between adenocarcinoma, squamous cell carcinoma, and normal lung tissue. Intriguingly, the program was trained to predict the 10 most commonly mutated genes in adenocarcinoma and found that six of them—STK11, EGFR, FAT1, SETBP1, KRAS, and TP53—can be predicted from pathology images, with AUCs from 0.733 to 0.856 as measured on a held-out population. The genetic changes identified by this study often cause the abnormal growth seen in cancer and they can change a cell’s shape and interactions with its surroundings, providing visual clues for automated analysis.

In another study, researchers used machine learning and retrospectively identified multiple factors that underlie cancer immunotherapy success which potentially allows better target immunotherapy treatment to those who will benefit [60]. To generate their computer model, researchers analyzed data (measured mutations and gene expression in the tumor and T cell receptor (TCR) sequences in the tumor and peripheral blood in urothelial cancers treated with anti-PD-L1) from 21 patients with bladder cancer from a clinical trial dataset of urothelial cancers from Snyder et al. [61] with a uniquely rich data set that captured information about tumor cells, immune cells, and patient clinical and outcome data. Instead of modeling the clinical response of each patient directly, researchers modeled the response of each patient’s immune system to anti PDL-1 therapy and used the predicted immune responses to stratify patients based on expected clinical benefit. Their computer model identified key features associated with a specific response to the drug (i.e. PD-L1 inhibitor) and applied 36 different features-multi-modal data set into their machine learning algorithm and allowed the algorithm to identify patterns that could predict increases in potential tumor-fighting immune cells in a patient’s blood after treatment. The machine learning algorithm identified 20 features. When they analyzed these features as a panel, they were able to describe 79 percent of the variation in patient immune responses. This suggested that the comprehensive set of features collected and analyzed for these patients may predict the patient immune response with high accuracy. However, if the researchers excluded any one of the three categories from the model (tumor data, immune cell data or patient clinical data) the algorithm can no longer predict immune response with high accuracy and confidence (the model could only predict at most 23 percent of the variation). Authors concluded that integrative models of immune response may improve our ability to predict the patient response to immunotherapy. However, this study only analyzed a small set of patient data (it only incorporated data from 21 patients, which is far too few to be predictive for the general population) and requires validation of this approach in a larger cohort of patients.

We also recently used a similar machine learning approach that enabled us to identify multiple factors that underlie short-term intensive insulin therapy (IIT) therapy success early in the course of type 2 diabetes which potentially allowed better targeted treatment to those patients who will benefit the most [23]. For that, we developed a model that could accurately predict the response to short-term intensive insulin therapy which provided insight into molecular mechanisms driving such response in humans. We selected a machine learning approach based on the random forests (RF) method, which implements an out-of-bag (“bagging”) technique to monitor error and ensure unbiased prediction with reduced risk of overfitting. For our analysis, the RF algorithm was implemented using the “randomForestpackage” in the R environment. As reported by [62], “by using bagging in tandem with random feature selection, the out-of-bag error estimate is as accurate as using a test set of the same size as the training set. Therefore, using the out-of-bag error estimate removes the need for a set aside test set.” In conclusion, our study identified potential responders to IIT (a current limitation in the field) and provided insight into the mechanism of pathophysiologic determinants of the reversibility of pancreatic islet beta-cell dysfunction in patients with early type 2 diabetes.

The advancements in digital health opportunities have also arisen numerous questions and concerns for the future of biomedical research and medical practice especially when it comes to reliability of AI-driven diagnostic tools, the impact of these tools on clinical practice and patients; vulnerability of algorithms to bias and unfairness, and ways to detect and improve the bias and unfairness in machine learning algorithms [63].

In summary, we hope that the AI program in a not too distant future helps to identify or even predict mutations instantly, avoiding the delays imposed by genetic tests, which can take weeks to confirm the presence of mutations. These findings suggest that AI and machine learning models can assist pathologists in the detection of cancer subtype or gene mutations in an efficient and expeditious way.

### Deep phenotyping—linking physiological abnormalities and molecular states—from bedside to bench

The analysis of phenotype plays a key role in medical research and clinical practice towards better diagnosis, patient stratification, and selection of best treatment strategies. In biology “phenotype” is defined as the physical appearance or biochemical characteristic of an organism as a result of the interaction between its genotype and the environment “Deep phenotyping” is defined as the precise and comprehensive analysis of phenotypic abnormalities in which the individual components of the phenotype (taking a medical history or a physical examination, diagnostic imaging, blood tests, psychological test, etc., in order to make the correct diagnosis) have been observed and described [64]. However, to understand the pathogenesis of a disease, several key points must be considered, such as the spectrum of complications, classification of patients into more homogeneous subpopulations that differ with respect to disease susceptibility, genetic and phenotypic subclasses of a disease, family history of disease, duration of disease, or to the likelihood of positive or adverse response to a specific therapy.

The concept of “PM” which aims to provide the best available medical care for each individual, refers to the stratification of patients into more homogeneous subpopulations with a common biological and molecular basis of disease, such that strategies developed from this approach is most likely to benefit the patients [Committee on the Framework for Developing a New Taxonomy of Disease, 2011]. A medical phenotype comprises not only the abnormalities described above but also the response of a patient to a specific type of treatment. Therefore, a better understanding of the underlying molecular factors contributing to disease and associated phenotypic abnormalities requires that phenotype is linked to molecular profiling data.

**Fig. 4:**
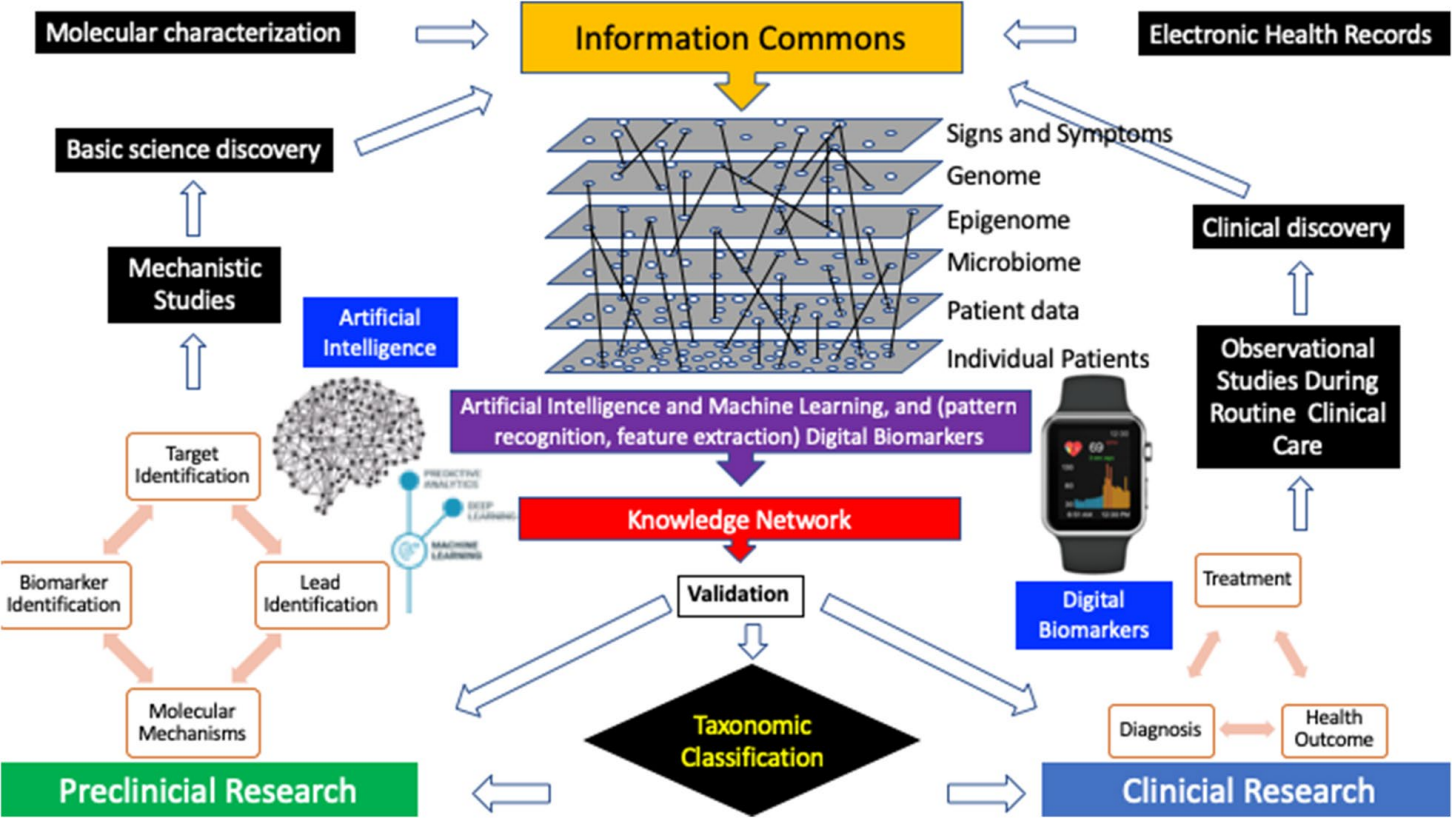
Schematic of a comprehensive biomedical knowledge network that supports a new taxonomy of disease. The knowledge network of disease would incorporate multiple parameters rooted in the intrinsic biology and clinical patient data originating from observational studies during normal clinical care feeding into Information Commons which are further linked to various molecular profiling data enabling the formation of a biomedical information network resulting in a new taxonomy of disease. Information Commons contains current disease information linked to individual patients and is continuously updated by a wide set of new data emerging though observational clinical studies during the course of normal health care. The data in the Information Commons and Knowledge Network provide the basis to generate a dynamic, adaptive system that informs taxonomic classification of disease. This data may also lead to novel clinical approaches such as diagnostics, treatments, prognostics, and further provide a resource for new hypotheses and basic discovery. At this intersection, artificial intelligence and machine learning may help to analyze this highly complex large dataset by pattern recognition, feature extraction yielding Digital BMs. Validation of the findings that emerge from the Knowledge Network, such as those which define new diseases or subtypes of diseases that are clinically relevant (e.g. which have implications for patient prognosis or therapy) can then be incorporated into the New Taxonomy of disease to improve diagnosis (i.e. disease classification) and treatment. This multi-parametric taxonomic classification of a disease may enable better clinical decision-making by more precisely defining a disease (adapted from [72])

Therefore, deep phenotyping, combined with advanced molecular phenotypic profiling such as genetics and genomics including Genome-wide association studies (GWAS), epigenetics, transcriptomics, proteomics, and metabolomics, with all their limitations, enables the construction of causal network models (Fig. 4) in which a genomic region is proposed to influence the levels of transcripts, proteins, and metabolites. This takes advantage of the relative (*i.e.* the function of regulatory RNAs and epigenetic modifications on phenotype) unidirectional flow of genetic information from DNA variation to phenotype.

As discussed by Schadt et al. [65] the relationships between various physiological phenotypes (e.g. physiological traits) and molecular phenotypes (*e.g.* DNA variations, variations in RNA transcription levels, RNA transcript variants, protein abundance, or metabolite levels) together constitute the functional unit which must be examined to understand the link to disease and strata of more homogeneous population representing the phenotype. All this can accelerate the identification of disease subtypes with prognostic or therapeutic implications, and help to develop better treatment strategies. Therefore, phenotypic analysis bears great importance for elucidating the physiology and pathophysiology of networks at the molecular and cellular level because it provides clues about groups of genes, RNAs, or proteins that constitute pathways or modules, in which dysfunction can lead to phenotypic consequences. Several recent studies have shown the utility of correlating phenotypes to features of genetic or cellular networks on a genome scale [66–69]. The emerging field of “Knowledge Engineering for Health” proposes to link the research to the clinic by using deep phenotypic data to enable research based on the practice and outcomes of clinical medicine which consequently lead to decision making in stratified and PM contexts [70].

### The knowledge network of disease

As illustrated in Fig. 4, and further discussed in the literature [71] a knowledge network of disease should integrate multiple datasets and parameters to yield a taxonomy heavily embedded in the intrinsic biology of disease. Despite physical signs and symptoms are the overt manifestations of disease, symptoms are often non-specific and rarely identify a disease with confidence and they are not as objective and not quantitative. In addition, a number of diseases— such as different types of cancer, cardiovascular disease, and HIV infection are asymptomatic in early stages. As a result, diagnosis based on traditional “signs and symptoms” alone carries the risk of missing opportunities for prevention, or early intervention.

On the other hand, advances in liquid biopsies, which analyze cells, DNA, RNA, proteins, or vesicles isolated from the blood as well as microbiomes have gained particular interest for their uses in acquiring information reflecting the biology of health and disease state. Biology-based BMs of disease such as genetic mutations, protein, metabolite BMs, epigenetic alterations of DNA, alterations in gene expression profiles, circulating miRNAs, cell-free DNAs, exosomes, and other biomolecules have the potential to be precise descriptors of disease.

When multiple BMs are used in combination with conventional clinical, histological, and laboratory findings, they often are a more accurate, sensitive, specific for the precise description and classification of disease.

In the near future, it is anticipated that comprehensive molecular profiling and characterization of healthy persons and patients will take place routinely as a normal part of health care even as a preventive measure prior to the appearance of disease, thus enabling the collection of data on both healthy and diseased individuals on a grander scale. The ability to conduct molecular characterizations on both non-affected and disease affected tissues would enable monitoring of the development and natural history of many diseases.

## Summary

The drug development is a challenging long process with many obstacles on the way. Though several strategies have been proposed to tackle this issue, there is a general consensus that a better use of BMs, omics data, AI and machine learning will accelerate the implementation of a new medical practice that will depart from the widely spread concept “one drug fits all”.

In conclusion, drug developers must combine traditional clinical data with patients’ biological profile including various omics-based datasets to generate an “information-based” model that utilizes complex datasets to gain insight into disease and facilitate the development of more precise, safer, and better-targeted therapies for a more homogeneous patient population.

### Review criteria

Publicly available information such as PubMed and Internet were used for the literature review. We focused on identifying articles published on the use of multiple technologies for the discovery and development of clinically relevant BMs, omics platforms, and other relevant topics in the subject area. The research was restricted to the most recent studies in this field and all research was limited to human studies published in English.

## Abbreviations

AD: Alzheimer’s Disease
Ag: antigen
AI: artificial intelligence
BMs: biomarkers
BETi: inhibitors of bromodomain and extra terminal proteins
Carbo: carboplatin
Cf-DNA: cell-free DNA
cf-RNA: cell-free RNA CSF1: colony stimulating factor 1
CDX: companion diagnostics
CFM: cyclophosphamide
CTC: circulating tumor cells
CTLA-4: cytotoxic T-lymphocyte–associated antigen 4
CTTI: The Clinical Trials Transformation Initiative
DNA: deoxyribonucleic acid
E.g.: exempli gratia
Etc.: etcetera
FDA: The Food and Drug Administration
FLS: fibroblast-like synoviocytes
GWAS: genome-wide association study
HDAC: histone deacetylase
HMA: hypomethylating agents
ICGC: International Cancer Genome Consortium (ICGC)
IDO: indoleamine 2,3-dioxyenase
I.E.: Id Est
IIT: intensive insulin therapy
LAG-3: lymphocyte-activation gene 3
LN: lymph nodes
MDSC: myeloid-derived suppressor cells
MHC: major histocompatibility complex
M&S: modeling and simulation
miRNAs: microRNAs
MS: multiple sclerosis
OA: osteoarthritis
IO: immuno-oncology
P13K: phosphoinositide 3-kinase
PD-1: programmed cell death-1
PD-L1: programmed death-ligand 1
PM: precision medicine
RA: rheumatoid arthritis
RF: random forests
RNA: ribonucleic acid
SLE: systemic lupus erythematosus
STING: stimulator of interferon genes
TCR: T cell receptor (TCR)
TIM3: T cell immunoglobulin and mucin domain 3
TLR: toll-like receptor
TILs: tumor-infiltrating lymphocytes
TME: tumor microenvironment
Treg: regulatory T cells
Wnt: wingless/integrated 1
